# Antibacterial and Antimycotic Activity of *Epilobium angustifolium* L. Extracts: A Review

**DOI:** 10.3390/ph16101419

**Published:** 2023-10-05

**Authors:** Mariola Dreger, Artur Adamczak, Joanna Foksowicz-Flaczyk

**Affiliations:** 1 Department of Biotechnology, Institute of Natural Fibres and Medicinal Plants—National Research Institute, Wojska Polskiego 71b, 60-630 Poznan, Poland; 2 Department of Breeding and Botany of Useful Plants, Institute of Natural Fibres and Medicinal Plants—National Research Institute, Kolejowa 2, 62-064 Plewiska, Poland; artur.adamczak@iwnirz.pl; 3 Department of Bioproducts Engineering, Institute of Natural Fibres and Medicinal Plants—National Research Institute, Wojska Polskiego 71b, 60-630 Poznan, Poland; joanna.flaczyk@iwnirz.pl

**Keywords:** willowherb, fireweed, *Chamerion angustifolium*, antibacterial activity, antifungal activity, minimum inhibitory concentration (MIC)

## Abstract

The aim of this work was to provide an overview of available information on the antibacterial and antifungal properties of *Epilobium angustifolium* extracts. A literature search of Scopus, PubMed/Medline, and Google Scholar for peer-reviewed articles published between January 2000 and June 2023 was undertaken. A total of 23 studies were eligible for inclusion in this review. Significant variation of antimicrobial activity depending on the tested species and strains, type of extract solvent, or plant organs utilized for the extract preparation was found. *E. angustifolium* extracts were active against both Gram-positive and Gram-negative bacteria and showed antimycotic effects against the fungi of *Microsporum canis* and *Trichophyton tonsurans* and the dermatophytes *Arthroderma* spp. Greater susceptibility of Gram-positive than Gram-negative bacteria to fireweed extracts was found. A strong antibacterial effect was recorded for *Staphylococcus aureus*, *Bacillus cereus*, *Micrococcus luteus*, *Escherichia coli*, *Klebsiella pneumoniae*, *Pseudomonas aeruginosa*, and *Acinetobacter baumannii* including multi-drug resistant strains. *E. angustifolium* extract might find practical application as an antimicrobial in wound healing, components of cosmetic products for human and animals, or as food preservatives.

## 1. Introduction

Recently, the expansion of drug-resistant pathogens has created demand for novel antimicrobials and stimulated the search for natural plant-based compounds as alternatives to synthetics [1,2,3]. Plants are a promising source of antimicrobial compounds: tannins, flavonoids, phenolic acids, essential oils, saponins, alkaloids, etc. Particular attention is paid to polyphenols due to the high diversity in their chemical structure and different mechanisms of activity. Therefore, polyphenol-rich species have been studied in search of new antimicrobials. In the last decade, the chemistry and biological activity of *Epilobium angustifolium* and related species have been studied intensively [4,5,6,7,8].

**Figure 1:**
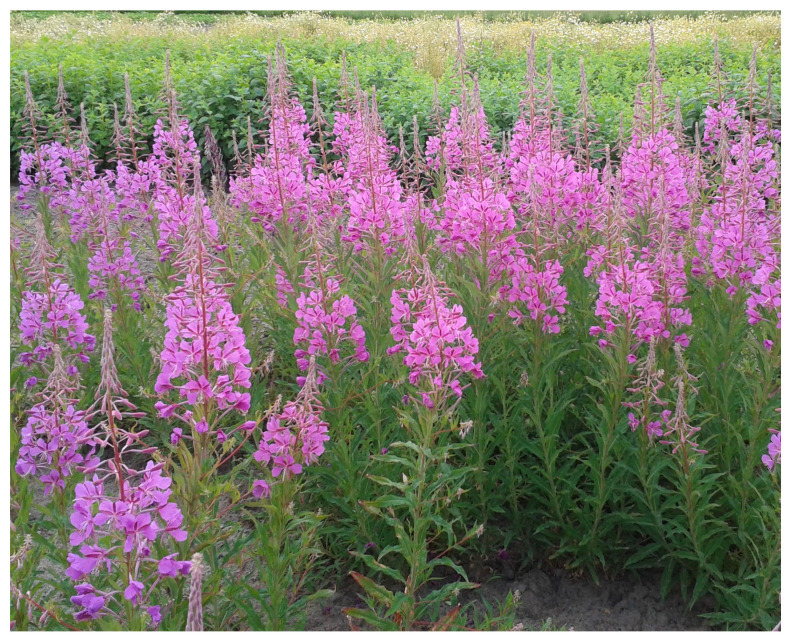
*Epilobium angustifolium* during blooming phase.

*Epilobium angustifolium* L. (fireweed or rosebay willow herb) is a well-known medicinal plant from the Onagraceae family (Figure 1). The species is distributed widely in the temperate zone of North America and Eurasia. Fireweed plants have been traditionally used as a remedy for various conditions including wound healing, infections, skin infections and diseases, colds, urinary problems such as benign prostatic hyperplasia (BPH) or prostatitis, gastric disorders, migraine headaches, and sleeping disorders [5,7,9]. Today, *Epilobii angustifolii herba* (herb) is often used as a component of nutraceuticals, diet supplements, and cosmetic products. Herb and extracts are commercially available for various indications including BPH, skin irritations, gastrointestinal disorders, or even prostate cancer [6]. The EMEA monograph on *E. angustifolium* and *E. parviflorum* stated that herbs of these species meet the requirements for “traditional use” as teas and infusions with indications for lower urinary tract symptoms related to BPH [10]. Efficacy of *E. angustifolium* in treatment of BPH has not been sufficiently proven. To date, only one clinical trial has been published [11]. The results of this randomized double-blind, placebo-controlled trial showed a decrease in the PVR (post-void residual), IPSS (International Prostate Symptom Score), and nocturia after intake of a food supplement containing standardized *E. angustifolium* extract. It should be stressed that a number of preclinical studies have documented anti-cancer [12,13,14], anti-androgen [15,16], anti-proliferative [17,18,19,20,21], anti-inflammatory [22,23,24], and antioxidant [25,26,27] properties of *E. angustifolium* extracts. Analgesics [28], anticholinesterase [23], and skin photoprotective activities of fireweed extract were also reported [29]. Recently, some studies also showed wound-healing [30] and cosmetic properties of fireweed [31,32,33].

The wide spectrum of biological activity of *E. angustifolium* extracts results from their complex and diverse chemical composition. More than 250 compounds have been identified, including: ellagitannins (hydrolysable tannins), flavonoids, phenolic acids, lignans, steroids, triterpenoids, fatty acids, essential oil, and alkaloids [7]. Medicinal properties of *E. angustifolium* were attributed to the synergic interactions of polyphenols and the high concentration of oenothein B—a macrocyclic (dimer) ellagitannin. Therefore, oenothein B and quercetin-3-*O*-glucuronide (flavonoid) have been proposed as marker compounds for standardization of the raw material [34]. Oenothein B is the most abundant ellagitannin in fireweed plants. This compound represents about 4–8% of the dry mass of herb depending on season, harvest time, plant organ, or genotype [35,36,37,38]. Oenothein B showed a broad spectrum of pharmacological properties including antioxidant, anti-cancer, anti-androgen, immunostimulatory, metal binding, and antimicrobial activities [39,40,41]. Apart from oenothein B, other ellagitannins from monomeric up to heptamers (e.g., tellimagrandin I, II, woodfordin, oenothein A, and others) were identified in *E. angustifolium* plants [42]. Tannins are known for their antibacterial properties because they react irreversibly with membrane proteins, neutralizing bacteria [43]. Several different mechanisms of action have been proposed for the antibacterial activity of tannins, such as: inhibition of extracellular microbial enzymes, oxidative phosphorylation, and disruption of cellular membrane permeability. Tannins bind to proteins through non-covalent bonds, leading to the morphological and structural changes and consequently to damages of the membrane integrity. Due to the diversity in the chemical structure of the compounds in this class, potentially each of them possesses antimicrobial properties.

Diverse flavonoids, particularly flavonol aglycones—quercetin, kaempferol, and myricetin—were identified in *E. angustifolium* herb. Flavonoids usually represent about 1–2% of the dry mass of plants, but in the mountains, their concentration can be higher, up to 4% [44]. Among flavonoids, quercetin-3-*O*-glucuronide is a dominant compound [4]. This substance has shown anti-inflammatory, neuroprotective, and nephroprotective activities [45,46,47]. The flavonoid distribution differs in flowers and leaves [36]. Flowers contain flavonoids with a rhamnose sugar moiety, in contrast to leaves, where these flavonoids are absent or rare; therefore, extracts prepared from leaves and flowers may differ in their activity. Quercetin-3-*O*-rhamnoside and a new compound, 1-(5-(hydroxymethyl) furan-2-yl)-6-methoxyisochroman-7-ol, have been identified as the most active contributors of anti-inflammatory activity of this species [24].

Phenolic acids are significant components of therapeutic activity of *E. angustifolium* extracts. Gallic, caffeic, ellagic, ferulic, and protocatechuic acids as well as caffeoylquinic acid isomers and others have been found [7,37]. Gallic acid was identified as the principal compound responsible for the antioxidant and therapeutic effect against BPH [16,26]. Phenolic acids have shown a wide range of biological activities including antibacterial, antiparasitic, and antiviral properties [48,49]. Therefore, these substances might affect or modulate the antimicrobial effect of extracts.

Essential oil constituents are known for their antimicrobial activity. The biological activity of the essential oil of *E. angustifolium* has not been thoroughly studied, but antioxidant, antibacterial, and antimycotic properties have been documented [50,51,52]. Other groups of active metabolites such as alkaloids (angustifoline A), lignans, fatty acids (tricosanoic, nervonic, linoleic, palmitic, caprylic, caproic, butyric, and others), and sterols (campesterol, stigmasterol, *β*-sitosterol, cholesterol, and their derivatives) have also been identified [13,53,54]

To date, *E. angustifolium* extracts have been tested in the treatment of BPH, in wound healing or as ingredients of cosmetic products, nano-bactericides [55], and as a food preservative [56]. Antimicrobial properties of fireweed have been studied since the beginning of this century, but in the last decade, the number of publications has increased significantly. However, the data are incomplete and scattered, so a new, comprehensive summary is needed. 

The aim of this study was to provide an overview of available information on the antibacterial and antifungal properties of *E. angustifolium* extracts. This work reviews the current state of knowledge and discusses antimicrobial activity and the relationship between the phytochemical composition of extracts.

## 2. Results

The selected literature includes twenty-three articles published between 2000 and 2023, although 13 of them were published in the last three years (2020–2023). The literature on *E. angustifolium* antibacterial and antifungal activity is scarce. Usually, fireweed among other rich-polyphenol species was tested to determine antimicrobial activity (Table 1). The gathered literature also includes the assessment of antimicrobial activity of hydrogels containing fireweed extracts [57,58,59,60,61,62,63,64,65,66,67,68,69,70,71,72,73] and bactericides with silver nanoparticles [55]. Extracts were prepared from different parts of plants: roots [57,62], leaves [56,60,64,74,75,76], flowering aerial parts [13,60,68,69,70,73,76], flowers [60,64,72], aerial parts or herb [55,58,59,65,71], the whole plant [63], and seeds [61]. In some articles [66,67,74], the plant parts were not specified. Water, ethanol, methanol, isopropanol, hexane, and dichloromethane were used as the solvents, but aqueous and ethanolic extracts were the most often tested. *E. angustifolium* extracts were prepared in different ways, e.g., using a shaker incubator and evaporator [72], by maceration of plant material with solvent and centrifugation [60], by evaporation and lyophilization [65], or by ultrasonication of infusion and lyophilization [56]. Phytochemical characterization of tested extracts was included in some articles [13,55,56,67,68,69,70,71,73,74,75,76], but it was usually limited to the total content of polyphenols and/or the total content of flavonoids and tannins. 

Different methods and assays such as the disc diffusion test, well diffusion method, cylinder diffusion method, broth dilution method, or quorum sensing assay [65] were applied for assessment of antimicrobial activity. In the case of probiotic bacteria, the effect of in vitro digested extract was measured using the optical density [74]. Twenty-six fungal and 39 bacterial species including 21 Gram-positive and 18 Gram-negative species were tested (Table 2 and Table 3). Various strains of *E. coli*, *S. aureus*, *P. aeruginosa*, and *C. albicans* were the most often tested. Standard strains, MDR [55,64], and clinically isolated strains [55,59,62,63] were also used. The anti-biofilm activity of extracts was tested using the wild type and the biomonitor strains of *Chromobacterium violaceum* [65]. The effect of extract on lactic acid bacteria strains was also determined [74,75]. The antibacterial effects of *E. angustifolium* extracts are summarized in Table 2. The antimycotic activity of extracts is presented in Table 3.

### 2.1. Antibacterial Activity

Screening of the activity of fireweed extracts revealed strong variation in the antimicrobial properties depending on the microbial species and strains. The MIC values ranged from 0.625 µg/mL to 16.2 mg/mL (Table 2). The lowest MIC value (0.625–1.25 µg/mL) was recorded for MDR strains of *S. aureus, E. coli*, *K. pneumoniae, A. baumannii*, and *P. aeruginosa* strains treated with nanoparticles synthesized with aqueous extract of *E. angustifolium* [55]. Very low MICs (<100 µg/mL) were also reported for *B. cereus* [67], *E. coli* [67,74,75], *P. aeruginosa* [67], and *K. pneumoniae* [59]. Significant differentiation in the MICs between strains was detected for *E. coli* (from 0.625 µg/mL to 16.2 mg/mL), *S. aureus* (from 0.625 µg/mL to 7.6 mg/mL), and *P. aeruginosa* (from 1.25 µg/mL to 9.1 mg/mL). Regarding the disc diffusion method, the best bactericidal effect (>20 mm) was recorded for *B. cereus* [68], *S. aureus* [55,72], *E. coli* [72], *K. pneumoniae*, and *P. aeruginosa* [66]. Moderate activity (bacteriostatic effect) was documented against *Enterococcus* sp., *Bacillus pseudomycoides*, *Staphylococcus* sp. (except some *S. aureus* strains), or *P. vulgaris*. Weak or no activity against *L. monocytogenes*, *S. enteritidis*, *Salmonella* sp., *Shigella flexneri*, or *Serratia* sp. was detected. Alcoholic (methanolic and ethanolic) and water extracts were the most active. Less effective were hexanoic [66] and dichloromethane extracts [75]. The different plant parts utilized for extract preparation demonstrated varied antibacterial effects. Extracts prepared from leaves and flowering parts of plants were equally active, but slightly better results were obtained for flowering parts [60]. Seed extract showed a moderate effect or was inactive [61].

### 2.2. Antifungal Activity

Root extracts demonstrated significant antifungal activity against *C. glabrata*, *C. lusitaniae* [62], and *M. gypseum* [57]. The effect was particularly strong against *M. gypseum* and comparable to berberine (positive control) [57]. Strong antimycotic properties of ethanol and water extracts were noted against *M. canis* [59] and *T. tonsurans* [67]. The recorded MIC values were the lowest: 10 µg/mL and 7.8–15.6 µg/mL, respectively. Water extract was effective against dermatophytes from *Arthroderma* genus, and the most sensitive species was *Arthroderma* *crocatum* (MIC = 19.68 µg/mL) [67]. Significant antimycotic activity was recorded against *T. rubrum* (MIC = 16–125 µg/mL), though the effect was strain-dependent [67]. The extracts demonstrated weak or moderate activity against *Candida* sp., but the effect was strongly dependent on species and strain. *C. tropicalis*, *C. maltosa*, and *C. glabrata* (clinically isolated strain) were more sensitive [62,67,75]. No activity was observed against *Aspergillus niger*, *A. flavum*, *A. fumigatus*, and *Fusarium* sp. [57,58,62].

## 3. Discussion

### 3.1. Antibacterial Activity

*E. angustifolium* extracts have shown strong variation in the biological activity depending on the bacterial species and strain. A strain-dependent antimicrobial effect is well documented, e.g., laurel activity against three *Salmonella* Typhimurium strains (4/74, FS8, FS115) [77] or the anti-biofilm effect of sage extract against *Listeria monocytogenes* [78]. Varied activity even against the same strains, e.g., *B. subtilis* ATCC 6633 [58,64,75], *E. faecalis* ATCC 29212 [70,73,76], or *S. aureus* ATCC 25923 [56,66,69,71], was recorded in individual studies. Aside from the strain effect, other factors such as different methods of assessment, methods of extract preparation, type of solvents, origin of the plant material, harvest time, etc., might generate different results and cause discrepancies between studies [79]. For the assessment of antimicrobial activity, the well variant of the microdilution method and the disc diffusion test were the most often chosen. Some authors used OD or IC_50_ methods [74,75]. However, the plant extracts were tested in various concentrations and often without detailed phytochemical characteristics. The quantity and quality of bioactive compounds in fireweed plants vary depending on the plant origin, season, phase of growth, harvest time, organs, or genotype [36,37,38,80]. Plant material used for extract preparation was obtained from a local market [66], herbal companies [56,63,67,74], and a nursery collection [75], or it was collected from a natural environment [13,55,65,68,69,70,71,73]. It should be added that plant material originated from different climatic zones and countries, e.g., Russia [55,72,75], Turkey [13,65,66,69,70], Poland [70,73,74], Ukraine [68], Finland [58], USA [61], and Canada [63].

Generally, a weak or moderate effect of seed extract was found [61]. Comparatively tested leaves and flower extracts demonstrated similarly weak activity against *E. coli* and *P. aeruginosa*, but a flower extract showed a stronger inhibitory effect against *S. aureus* (11 mm vs. 17 mm) and *C. albicans* (15 mm vs. 20 mm) [60]. Kosalec et al. 2013 obtained similar and consistent results [64]. Both extracts (leaves and flowers) were active against Gram-positive bacteria, but leaf extract had a significantly lower MIC value (9.1 vs. 16.2 mg/mL) against the *C. albicans* strain. The differences in the extracts’ activity might have resulted from their different chemical compositions. Diversity in the organ distribution of the main phenolic compounds in flowers and leaves of *E. angustifolium* was found by Baert et al. 2017 [36]. Hexameric and heptameric ellagitannins were 3–4 times more abundant in flowers than in leaves. Flavonoids such as quercetin-3-*O*-rhamnoside, myricetin-3-*O*-rhamnoside, and kaempferol-3-*O*-rhamnoside were specific to flower tissue and were absent from leaves. Therefore, the different compositions of ellagitannins and flavonoids in flowers and leaves might be responsible for the varied antimicrobial effect. 

The other crucial factor is the solvent used for extraction [81,82,83]. Polarity of the solvent is a principal issue due to the different solubility of the various classes of antimicrobial compounds in the solvents [84]. Decreasing antimicrobial activity of methanolic > ethanolic > hexanoic > water extract against *E. coli*, *S. aureus*, *K. pneumoniae*, *P. aeruginosa* was reported by Güven at al. 2020 [66]. In another study, a dichloromethane extract containing non-polar compounds showed no or little activity, but methanolic and ethanolic extracts were both active [75]. Alcoholic extracts are typically rich in medium-polar compounds such as tannins and flavonoids. Good antimicrobial activity of tannins and flavonoids has been documented [85,86] and supports the obtained results. On the other hand, despite quantitative and qualitative differences in flavonoid composition and phenolic acid content in methanolic and ethanolic extracts, both extracts showed similar antibacterial and antioxidant activity [13]. 

*E. angustifolium* extracts were active against both Gram-positive and Gram-negative bacteria. Lower activity against Gram-negative than Gram-positive bacteria was found [56,63,64,68,70,71,73]. This susceptibility of Gram-positive bacteria has often been observed in antimicrobial studies on plant extracts, e.g., sage [87], *Artemisia vulgaris* [88], *Eucalyptus grandis* [89], etc., and it is attributed to the structure of the cellular membrane. Gram-negative bacteria have an outer membrane that restricts diffusion of compounds through its lipopolysaccharide wall and makes them more resistant to natural compounds or antibiotics [90,91].

Among Gram-positive bacteria, a high antibacterial effect expressed as a low MIC value (<300 µg/mL) or as the inhibition zone (>20 mm) was recorded for *S. aureus* [55,67,69,72] and *B. cereus* [67,68]. It is worth noting that tested extracts were effective against clinically isolated strains of *S. aureus* [63] including MRSA strains [55] and *M. luteus* [63]. Methicillin-resistant *Staphylococcus aureus* (MRSA) strains cause serious and mortal infections in the elderly and in immunocompromised patients [92,93]. Therefore, MRSA strains are considered as a very urgent health problem and in 2017, the WHO posted MRSA on a list of global priority pathogens classified as high priority (priority 2) [94]. According to the literature, the leaf extracts of *Polyalthia longifolia* [95], *Xylopia pancheri* [96], *Cistus salviifolius*, and *Punica granatum* [91], and other species demonstrated antibacterial properties against clinical isolate strains of *S. aureus* including MRSA. Interestingly, punicalagin (ellagitannin) and ellagitannin-rich extracts from *C. salviifolius* and *P. granatum* were the most active against 100 *S. aureus* (50 MRSA) clinical isolates [97]. In another study, ellagitannins such as salicarinin A, rugosin D, casuarictin, tellimagrandins I and II, pentagalloylglucose, stachyurin, casuarinin, vescalagin, castalagin, rugosin E, sanguiin H-6, and lambertianin C significantly inhibited growth of methicillin-susceptible strains of *S. aureus* [98]. Regarding diversity and high concentration of ellagitannins in *E. angustifolium* ethanolic and aqueous extracts, this group of secondary metabolites might be responsible for the anti-staphylococci effect. However, detailed phytochemical studies are needed to make definitive conclusions.

Another Gram-positive bacterial species, *Micrococcus luteus*, was sensitive to fireweed extracts [63]. *E. angustifolium* extract inhibited the growth of *M. luteus* more effectively than vancomycin or tetracycline (250 µg/mL). Unfortunately, the extract was not chemically studied, and no pure compounds were comparatively evaluated. Therefore, it is difficult to claim what active compound or class of secondary metabolite was responsible for this antibacterial effect in this case. It is worth noting that the digested extracts tested on probiotic bacteria (*Lactobacillus* and *Bifidobacterium* strains) did not inhibit the growth of these beneficial intestinal bacteria [74]. This is important in the context of fireweed plants’ utilization in herbal dietary supplements or teas.

Among Gram-negative bacteria, the best results (<300 µg/mL; >20 mm) were recorded for *E. coli* [55,67,69,72,74,75], *K. pneumoniae* [55,59,66], *P. aeruginosa* [55,59,66,67] *A. baumannii* [55], and *S.* Typhi [67]. *E. coli* strains showed varying sensitivity to *E. angustifolium* extracts. Generally, the fireweed extracts showed moderate activity against *E. coli*. The best effect was observed for the crude whole plant extract [63], which inhibited the growth of bacteria in culture more effectively than vancomycin and tetracycline (250 µg/mL). However, the authors pointed out that the limitation of their study was lack of a dose response determined for select dilutions of the crude plant extract and lack of phytochemical analysis of the extract. 

A methanolic extract of *E. angustifolium* also showed significant activity against *P. aeruginosa* and *K. pneumoniae* with a 23–25 mm inhibition zone mainly due to the properties of the solvent [66]. Aqueous and ethanolic extracts were less effective against the same strains tested in this study. 

*A. baumannii* and *P. aeruginosa* are pathogens with an urgent need for new antimicrobials, and their carbapenem-resistant strains are included in the WHO list of global priority pathogens classified as critical priority [94]. High activity against these clinically isolated members of ESKAPE drug-resistant pathogens was noted using bio-hybridized nanocellulose films which consist of silver nanoparticles synthesized with aqueous extract of *E. angustifolium* [55]. Among the test pathogens, methicillin-resistant *S. aureus*, *K*. *pneumoniae*, and *E. coli* strains were most sensitive (MIC = 0.625 µg/mL). The lowest activity was observed against *A. baumannii* strains (MIC = 1.25 µg/mL). The literature provides evidence of the effectiveness of biocidal nanomaterials against resistant bacteria [99,100]. Nano silver hybridized onto bacterial cellulose films showed significant activity against *E. coli* and *S. aureus* [101], or *S. aureus* and *P. aeruginosa* [102]. Green synthesized nanoparticles from *Plantago major* [103], *Prunus africana*, *Camelia sinensis* [104], and *Tradescantia pallida* extracts showed significant antimicrobial effects against *S. aureus*, *E. coli*, and *P. aeruginosa* [105]. In another study, the MIC values of green synthesized silver nanoparticles against *S. typhimurium* and *Y. enterocolitica* were 6.2 μg/mL and 3.1 μg/mL, respectively [106]. It is worth noting that the MIC/MBC values were significantly lower than those of antibiotics against tested pathogens. In this context, the low MIC values against MDR pathogens obtained by Baker et al. [55] seemed to be very promising and have potential for application in the biomedical sector. The safety issue of nanoparticles is still under discussion, but green synthesis has been regarded as safer, more environmentally friendly, non-toxic, and effectively scaled up for large-scale synthesis [107]. 

### 3.2. Antifungal Activity

*E. angustifolium* extracts showed strong antifungal properties against the dermatophytes *M. canis* [59] and *T. tonsurans* [67] with the MIC values of 10 µg/mL and 7.8–15.6 µg/mL, respectively. It should be noted that the MIC value of 3 μg/mL is considered as a concentration limit for the effectiveness of drug therapy in *M. canis* or *T. rubrum* in humans [108]. Significant antimycotic activity was also recorded against *T. rubrum*, *T. mentagrophytes*, and *Arthroderma* spp., with *A. crocatum* being the most sensitive species [67]. In contrast to dermatophytes, the *E. angustifolium* extracts were inactive or moderately active against *Candida* spp. with the exception of *C. tropicalis* [67] and *C. maltose* [75]. 

The resistance of fungal species, i.e., *Aspergillus* and *Candida* spp., is well documented [109], whereas in other dermatophyte species, it is rather seldom reported, but is verified in *T. rubrum*, *T. mentagrophytes* [110,111], and *M. canis* [112,113,114]. Therefore, plants that possess antidermatophytic properties might be potent as selective inhibitors of fungal activity and have economic value. It is worth noting that in another study on the related species *Epilobium parviflorum*, the average MIC values against the eight tested strains of *T. mentagrophytes* isolated from patients was 9.25 mg/mL [115]. In this regard, *E. angustifolium* extracts demonstrated much stronger activity, expressed in the MIC value range of 62.3–125 µg/mL [67]. 

*Cymbopogon* spp., *Eucalyptus robusta*, *Punica granatum*, and *S. baicalensis* are plant species with strong antifungal properties with an MIC value range of 12.5–100 μg/mL [116]. The main active compounds with proven antifungal activities are thymol, carvacrol, resveratrol, wogonin and other flavonoids (catechin), alkaloids (e.g., berberine, magnoflorine), gallic acid and other polyphenols such as tannins, terpenoids, and saponins. Gallic and ellagic acids as well as tannins have shown antifungal activity in numerous studies [85,117,118]. These compounds are natural constituents of *E. angustifolium* extracts and contribute to the antifungal and antibacterial activities. However, the exact antifungal components of *E. angustifolium* extracts and their mechanism of action are still unknown. 

### 3.3. Active Compounds of E. angustifolium Extracts and Mechanisms of Action

Most authors have attributed the antimicrobial activity of fireweed extracts to their rich polyphenol composition [13,56,64,67,69,71,76]. The antimicrobial properties of polyphenolic compounds are linked to their absorption by the bacterial membranes, which leads to membrane perforation and leakage of cellular contents [85]. The effect depends on the composition and concentration of phenolic compounds and their interactions. Polyphenols are also the main factors responsible for apoptotic and cytostatic activity of *E. angustifolium* extracts [12,14,119] as well as DNA binding properties [13]. Recently, Perużyńska et al. 2023 [14] tested the ethanolic extract of *E. angustifolium* on different lines of cancer cells using bacterial cellulose membranes as the matrix for the controlled delivery of the plant extract. The SEM analysis confirmed the deposition of active compounds inside the membranes and the cytotoxicity effect of the extract was dose and time-dependent. In other words, they confirmed that *E. angustifolium* extract was able to efficiently penetrate the bacterial membranes and induce apoptosis in the cancer cells.

Neumann et al. 2022 [55] tested 14 plant extracts and found a significant correlation between the antimicrobial activity and tannin yield in the extract, but not flavonoids. The *E. angustifolium* leaf extract, rich in tannins (10.41%) and flavonoids (1.9%), was the most active against yeast (e.g., *Candida maltose*) and *S. aureus.* In this study, the anti-biofilm effect of plant extracts against MDR *E. coli* strains was also investigated. *Polygonum bistorta* extract was most active (MIC 10 µg/mL), whereas *E. angustifolium* extract showed moderate activity (50–100 µg/mL). The result of correlation analysis of anti-biofilm activity and tannin content was moderate but statistically significant, but no correlation between flavonoids and anti-biofilm activity was found. This was surprising, because flavonoids are biofilm-inhibiting compounds. The authors explained that the assay used to evaluate biofilm inhibition did not reflect the full potential of the flavonoids and in the applied method, flavonoid content was calculated as hyperoside, which has comparatively low anti-biofilm activity. Regardless of this study, tannins and flavonoids are considered as the main contributors of antimicrobial activity [120,121,122]. 

Tannins might inhibit bacterial growth using different mechanisms such as inhibition of cell wall synthesis, iron chelation, disruption of the cell membrane, or inhibition of the fatty acid biosynthetic pathway [123]. The biological function of ellagitannins in plants is defensive, with an anti-herbivore role [124,125]. Their bioactivity can be attributed to the increasing number of hydroxyl groups present in the chemical structure, and in particular to the pyrogallol-type (1,2,3-trihydroxyphenyl) motif of the galloyl and galloyl-derived groups as well as to the substitution pattern and size of the ellagitannins [39,124]. The size of molecules and oligomeric linkage of ellagitannins might influence the antimicrobial activity. Dimers with an m-DOG linkage exhibited stronger inhibition than ellagitannin oligomers with m-GOD or m-GOG linkages [98].

The antibacterial effect of ellagitannins against *Staphylococcus aureus*, *Escherichia coli*, and *Clostridium perfringens* was documented [98]. The effect was the greatest against *S. aureus* and the weakest for *C. perfringens*. Salicarinin A and rugosin D inhibited the growth of *S. aureus* completely at a 0.5 mM concentration. Tellimagrandin II, rugosin D, agrimoniin, sanguiin H-6, and lambertianin C were the most efficient compounds against *E. coli*. The antibacterial effect against *S. aureus* was not clearly related to the molecular size or flexibility of ellagitannins; however, rugosins E and D and pentagalloylglucose with four or five free galloyl groups demonstrated stronger activity than the other ellagitannins with glucopyranose cores. The oligomeric linkage of ellagitannin and the number of free galloyl groups in pentagalloylglucoses were suggested as crucial factors in activity against *E. coli*. In turn, the antimicrobial effect against *C. perfringens* was attributed to the molecular size of ellagitannins. It should be added that macrocyclic ellagitannins such as oenothein B or A were not tested in this study. Oenothein B demonstrated antimicrobial properties against *Helicobacter pylori*, *Staphylococcus aureus* as well as fluconazole-sensitive and -resistant *Candida albicans*, *C. tropicalis*, and *C. glabrata* strains [126,127,128]. Molecular docking analysis proved that oenothein B interacted with specific amino acid residues located at the active site of *S. aureus* lactoferrin, whereas the phenolic hydroxyl groups were not associated with lactoferrin [128]. 

The antimicrobial activity of fireweed extracts was also related to the presence of flavonoids, particularly to quercetin and kaempferol [64,71,74]. Some authors attributed the antibacterial effect to the presence and the concentration of hydroxycinnamic acids [68,70,71].

Quercetin and its derivatives, which are the predominant compounds among *E. angustifolium* flavonoids, are known to have antibacterial and antiviral properties [129,130]. In turn, myricetin-3-*O*-rhamnoside (myricitrin) was effective against *B. cereus*, *E. coli*, *S. aureus*, and *K. pneumoniae* [131,132]. Flavonoids act via various mechanisms including suppression of nucleic acid synthesis, cytoplasmic membrane and energy metabolism, disturbances in adhesion and biofilm formation, inhibition of cell envelope synthesis, or membrane disruption [122,133,134]. The antibacterial effect of flavonoids is linked to their chemical structure, particularly to the presence, number, and position of functional groups: hydroxyl, methoxy, halogen, and methyl in two aromatic rings A and B [134].

Significant antimycotic properties of gallic, ellagic, and chlorogenic acids have been mentioned above. However, antibacterial activity against methicillin-resistant *S. aureus* [135], MDR *E. coli* strains [136], and *H. pylori* [137] has also been reported. Recently, ellagic and gallic acids showed antibacterial potential against clinical IBD (inflammatory bowel diseases) isolates [117]. 4-*O*-caffeolyqunic, neochlorogenic, and chlorogenic acids were identified as the most potent anti-inflammatory constituents of *E. angustifolium* extract [24]. Chlorogenic acid is an effective antimicrobial against both Gram-positive and Gram-negative bacteria [138,139,140]. The antimicrobial mechanism of activity of chlorogenic acid is explained by disruption of intracellular and outer membranes and consequently cell metabolism [141,142]. A more recent study indicates that chlorogenic acid may induce its action via downregulating ribosomal subunits, affecting lipid metabolism, and scavenging intracellular ROS [139]. 

The antibacterial activity of extracts might also be modulated and affected by the presence of other compounds. Notably, *C. salviifolius* extract was more effective against MRSA isolates, whereas *P. granatum* extract was more active against methicillin-sensitive *S. aureus* (MSSA) isolates [97]. Both extracts were abundant in hydrolysable tannins but *C. salviifolius* extract contained more flavonoids (myricetin and quercetin derivates), phenolic acids and coumarins. *E. angustifolium* plants are a rich source of hydrolysable tannins and flavonoids. Considering the chemical composition of *E. angustifolium* extracts and the antimicrobial potential of their constituents, the synergistic mechanism of action should be suggested rather than the activity of a certain compound at high concentration.

## 4. Materials and Methods

A systematic literature search of the electronic databases of Scopus, PubMed/Medline, and Google Scholar was conducted for peer reviewed articles to find studies focusing on antibacterial and antifungal properties of *Epilobium angustifolium* L. extracts. The time range was set between January 2000 and June 2023. In all searches, the following terms were used: “*Epilobium angustifolium*”/“*Chamerion angustifolium*”/“*Chamaenerion angustifolium*” and “antibacterial”/“antifungal”/“medicinal plants with antibacterial properties”. The search was not limited to title, keywords, and abstract, but the text of the articles was also explored. The obtained records of databases were further explored for articles that may match the search criteria. Studies were considered if they met the following criteria: (1) plant species—*Epilobium angustifolium*, (2) extracts prepared from plant material (roots, leaves, aerial parts, flowering aerial parts, seeds), (3) antibacterial or antifungal studies, (4) article written in English. Essential oils, honey, food supplements, and cosmetic products were excluded. The literature search yielded the following number of records: Scopus (29), PubMed/Medline (79), and Google Scholar (123). Reviews, conference proceedings, and articles concerning biological activities of *E. angustifolium* extracts without antimicrobial activities were not included. After rigorous selection, removing duplicates and irrelevant articles, only 23 articles were considered for this review (Table 1). 

## 5. Conclusions

The reviewed literature showed a broad spectrum of antimicrobial activity of *E. angustifolium* extracts and provided scientific evidence for the traditional utilization of this species. The studies demonstrated significant variation of antimicrobial activity depending on the tested species and strains, type of extract solvent, or plant organs utilized for the extract preparation. *E. angustifolium* extracts demonstrated antibacterial activity against both Gram-positive and Gram-negative bacteria and showed antimycotic effects against the fungi of *M. canis* and *T. tonsurans* and the dermatophytes *Arthroderma* spp. Greater susceptibility of Gram-positive than Gram-negative bacteria to fireweed extracts was found. A strong antibacterial effect expressed in the low MIC values (<300 µg/mL) or as in inhibition zones (>20 mm) was recorded for *S. aureus*, *B. cereus*, *E. coli*, *K. pneumoniae*, *P. aeruginosa, E. coli, S.* Typhi, and *A. baumannii* including methicillin-resistant strains. The antimicrobial activity of fireweed is attributed more to the rich polyphenol composition of extracts and their synergic interactions than the activity of a certain compound at a high concentration. Although the standardization of antimicrobial studies and the methods of chemical analysis of extracts have improved over the last decade, there is still a need for further improvement of methodological quality and comparative studies including isolated substances. Another challenge is the lack of adequate knowledge on the compositions and mechanisms of action of extracts. Nevertheless, the interesting results of the cited studies suggest potential utilization of fireweed extracts as antimicrobials in wound healing, components of cosmetic products for humans and animals, or a food preservative.

## Figures and Tables

**Table 1:** Studies testing antimicrobial activity of *Epilobium angustifolium* extracts.

No.	Plant Material/ Extractant	Experimental Method	Microbial Species	Effect	Reference
1	Roots/EtOH, H_2_O-EtOH (1:1)	Disk diffusion method	*Aspergillus fumigatus*, *Candida albicans*, *Cryptococcus neoformans*, *Microsporum gypseum*, *Saccharomyces cerevisiae*, *Trichophyton mentagrophytes*	Great antifungal activity against *M. gypseum*, *T. mentagrophytes*, *S. cerevisiae*, and *C. albicans*. The effect comparable to or even stronger than the positive control (berberine).	[57]
2	Aerial parts/80% MeOH	Cylinder diffusion method	*Aspergillus niger*, *Candida albicans*, *Escherichia coli*, *Staphylococcus aureus*	Significant antibacterial effect against *E. coli* and *S. aureus*. Slight or lack of antifungal activity against *C. albicans* and *A. niger*.	[58]
3	Aerial parts EtOH	Broth microdilution method (MIC, MCC)	*Bacillus subtilis*, *Candida albicans*, *C. glabrata*, *C. krusei, Enterococcus faecalis*, *Escherichia coli*, *Klebsiella pneumoniae*, *Listeria monocytogenes*, *Microsporum canis*, *M. gypseum*, *Pseudomonas aeruginosa*, *Salmonella enteritidis*, *Shigella flexneri*, *Staphylococcus aureus*, *Streptococcus pyogenes*, *S. sanguis*, *Trichophyton mentagrophytes*, *T. rubrum*	Very strong antifungal activity against *Microsporum canis* and strong antibacterial effect against *K. pneumoniae*.	[59]
4	Leaves, flowers, flowering aerial parts/H_2_O-EtOH (80:20)	Disk diffusion method	*Candida albicans*, *Escherichia coli*, *Pseudomonas aeruginosa*, *Staphylococcus aureus*	Activity of leaf and flowering part extracts against all microorganisms tested. The strongest effect against *C. albicans* and *S. aureus*.	[60]
5	Seeds/50% MeOH	Disk diffusion method	*Candida albicans*, *Escherichia coli*, *Pseudomonas aeruginosa*, *Staphylococcus aureus*	Poor activity only against *S. aureus*.	[61]
6	Roots/H_2_O	Broth microdilution method (MIC)	*Aspergillus flavus*, *A. fumigatus*, *Candida albicans*, *C. glabrata*, *C. krusei*, *C. lusitaniae*, *C. parapsilosis, C. tropicalis, Cryptococcus neoformans*, *Epidermophyton floccosum*, *Fusarium solani*, *Microsporum canis*, *Rhizopus* sp., *Saccharomyces cerevisiae, Trichophyton mentagrophytes*, *T. rubrum*, *T. tonsurans*	Strong antifungal effect, especially against *C. glabrata*, *C. lusitaniae*, and *S. cerevisiae* strains.	[62]
7	Whole plant/crude extract (Fytokem, SK, Canada)	Broth microdilution method (absorbance measurement)	*Escherichia coli*, *Micrococcus luteus, Pseudomonas aeruginosa, Staphylococcus aureus*	Activity against all bacteria tested. More effective than tetracycline against Gram-negative bacteria: *E. coli* and *P. aeruginosa*.	[63]
8	Leaves, flowers without stems/96% EtOH	Disk diffusion method, Broth microdilution method (MIC), Cell viability test (*C. albicans*)	*Bacillus subtilis*, *Candida albicans*, *C. dubliniensis*, *C. tropicalis*, *Escherichia coli*, *Proteus mirabilis*, *Pseudomonas aeruginosa*, *Saccharomyces cerevisiae*, *Staphylococcus aureus*	Weak antimicrobial activity, usually similar for both types of extracts (leaves and flowers).	[64]
9	Aerial parts/75% MeOH	Disk diffusion method, Quorum sensing (QS) inhibition activity assay	*Chromobacterium violaceum*	Dose-dependent QS inhibition activity. Reduction of violacein production by 41% at 250 μg/mL and 57% at 500 μg/mL concentration of plant extract.	[65]
10	Flowering aerial parts/EtOH, MeOH	Well diffusion method, Broth microdilution method (MIC, MBC)	*Bacillus cereus*, *B. subtilis*, *Candida albicans*, *C. krusei*, *C. tropicalis*, *Enterococcus faecalis*, *E. hirae*, *Escherichia coli*, *Klebsiella pneumoniae*, *Pseudomonas aeruginosa*, *Proteus vulgaris*, *Salmonella* Typhimurium, *Staphylococcus aureus*	Moderate antibacterial activity against *B. cereus* and *B. subtilis*, low—against *S. aureus* and *P. vulgaris*. No antifungal effect against *Candia* spp.	[13]
11	Local market, Turkey/99% EtOH, H_2_O, 95% HE, 99.7% MeOH	Disk diffusion method	*Escherichia coli*, *Klebsiella pneumoniae*, *Pseudomonas aeruginosa*, *Staphylococcus aureus*	Broad differentiation between antibacterial activity of particular extracts. The greatest sensitivity of *P. aeruginosa*, *K. pneumoniae*, and *S. aureus* to MeOH extract, while *E. coli* to EtOH and MeOH extracts.	[66]
12	OMEGA PHARMA Srl (Cantù, Italy)/H_2_O	Broth microdilution method (MIC)	*Arthroderma crocatum*, *A. currey*, *A. gypseum*, *A. insingulare*, *A. quadrifidum*, *Bacillus cereus*, *B. subtilis*, *Candida albicans*, *C. parapsilosis*, *C. tropicalis*, *Escherichia coli*, *Pseudomonas aeruginosa*, *Salmonella* Typhi, *Staphylococcus aureus*, *Trichophyton erinacei*, *T. mentagrophytes*, *T. rubrum*, *T. tonsurans*	Strong antimicrobial activity, especially against *T. tonsurans*, *T. rubrum*, *A. crocatum*, *A. currey*, *A. insingulare*, *C. tropicalis*.	[67]
13	Flowering aerial parts/no data	Well diffusion method, Broth microdilution method (MIC, MBC)	*Bacillus cereus*, *Candida* spp., *Escherichia coli*, *Staphylococcus aureus*	Strong antibacterial activity of 30 and 50% solution of extract against *B. cereus* and *S. aureus*.	[68]
14	Flowering aerial parts/EtOH	Disk diffusion method, Broth microdilution method (MIC)	*Escherichia coli*, *Salmonella* Typhimurium, *Staphylococcus aureus*	Low antibacterial effect compared to ciprofloxacin (disk diffusion method), while significant activity determined by the broth microdilution method.	[69]
15	Flowering aerial parts/70% EtOH	Well diffusion method	*Bacillus pseudomycoides*, *B. subtilis*, *B. thuringiensis*, *Enterococcus faecalis*, *E. faecium*, *Pseudomonas aeruginosa*, *P. fluorescens*, *Serratia lutea*, *S. marcescens*, *Streptococcus pneumoniae*	Strong antibacterial activity against *S. lutea*, *S. marcescens*, as well as *B. pseudomycoides* and *B. subtilis*.	[70]
16	Aerial parts/H_2_O, MeOH	Disk diffusion method	*Enterobacter cloacae, Escherichia coli*, *Klebsiella pneumoniae, Proteus vulgaris, Pseudomonas aeruginosa*, *Salmonella* Typhimurium, *Serratia marcescens, Staphylococcus aureus, S. epidermidis, Streptococcus pyogenes*	Strong antibacterial activity against *S. aureus, S. epidermis, S. pyogenes,* and *P. vulgaris*.	[71]
17	Flowers/H_2_O	Well diffusion method, Broth microdilution method (MIC, MBC)	*Candida albicans, Escherichia coli*, *Staphylococcus aureus*	Strong antimicrobial activity against *S. aureus, E. coli,* and *C. albicans*.	[72]
18	Flowering aerial parts/70% EtOH, 70% iPrOH, H_2_O in hydrogels	Well diffusion method	*Bacillus subtilis, B. pseudomycoides, Enterococcus faecalis, E. faecium, Escherichia coli, Pseudomonas fluorescens, Sarcina lutea, Serratia marcescens, Staphylococcus aureus*, *Streptococcus epidermidis, S. pneumoniae*	Moderate activity against *S. pneumoniae*, *E. faecalis*, *E. faecium, S. lutea*, and *E. coli*.	[73]
19	No data/infusion	In vitro digested infusion model. Optical density (OD600) with a Bioscreen C system	*Bifidobacterium adolescentis*, *B. longum, Enterobacter cloacae, Enterococcus faecalis, Escherichia coli, Lactobacillus rhamnosus*	No growth inhibition of *L. rhamnosus*, *Bifidobacterium* sp. contrary to *E. coli*.	[74]
20	Leaves/DCM, MeOH, H_2_O	Disk diffusion method, Anti-biofilm assay	*Bacillus subtilis, Candida maltosa, Escherichia coli* (including strains forming biofilms), *Pseudomonas aeruginosa*, *Staphylococcus aureus*	Strong antimicrobial activity against *C. maltose,* significant—against *S. aureus, E. coli, P. aeruginosa*. Poor or no activity of DCM extract. Weak or moderate anti-biofilm activity.	[75]
21	Leaves/H_2_O	Broth microdilution method (MIC)	*Bacillus cereus, B. subtilis, Clostridium sporogenes, Enterococcus faecalis, Escherichia coli, Lactobacillus rhamnosus, L. plantarum, Listeria innocua, L. monocytogenes, Salmonella* Hofit, *S. enterica*, *Staphylococcus aureus*	Significant activity against *S. aureus*, *L. monocytogenes, Bacillus* spp., *Lactobacillus* spp. No activity against Gram-negative bacteria. Resistance of *P. aeruginosa*.	[56]
22	Leaves, flowering aerial parts/30% EtOH	Disk diffusion method, Broth microdilution method (MIC)	*Bacillus cereus, Candida albicans, Enterococcus faecalis, Escherichia coli, Listeria monocytogenes, Pseudomonas aeruginosa, Salmonella enteritidis, Staphylococcus aureus*	Moderate activity against *B. cereus* and *E. faecalis.* No activity against Gram-negative bacteria.	[76]
23	Aerial parts/H_2_O/silver nitrate nanoparticles	Well diffusion method, Broth microdilution method (MIC)	*Acinetobacter baumannii*, *Escherichia coli, Klebsiella pneumoniae, Pseudomonas aeruginosa*, *Staphylococcus aureus* (MRSA)	Strong antibacterial effect against MRSA. Significant activity against *A. baumannii* in well-diffusion assay.	[55]

Abbreviations: DCM—dichloromethane; EtOH—ethanol; HE—*n*-hexane; MeOH—methanol; iPrOH -isopropanol; MCC—minimum cytocidal concentration; MIC—minimum inhibitory concentration; MDR—multi-drug-resistant; MRSA—methicillin-resistant *Staphylococcus aureus*.

**Table 2:** Antibacterial activity of *Epilobium angustifolium* extracts.

Genus	Species	Strains	Inhibition Zone	MIC/OD
*Bacillus*	*cereus*	PeruMycA 4		62.5–125 µg/mL [67]
		NCTC 74	17.0–25.0 mm [68]	30% solution [68]
		ATCC 11778	13.8–14.1 mm [76]	0.25% solution [56], MIC_50_ = 1/64 (aerial parts), MIC_50_ = 1/128 (leaves) [76]
		NRRL B-3711	activity found; no data [13]	1.56 mg/mL [13]
	*pseudomycoides*	not specified	6.0–11.5 mm [70], 10.0–13.0 mm [73]	
	*subtilis*	ATCC 6633	13.5 mm [64], activity found; no data [13]	325 µg/mL [59], 1.56 mg/mL [13], 4.6–6.1 mg/mL [64], 0.25% solution [56]
		PeruMycA 6		125–250 µg/mL [67]
		ATCC 6059	no activity [75]	0.25% solution [56]
		not specified	6.5–11.0 mm [70], 6.5–8.5 mm [73]	
	*thuringiensis*	not specified	5.5–9.0 mm [70]	
*Clostridium*	*sporogenes*	ATCC 11437		no activity at 0.5% solution [56]
*Bifidobacterium*	*adolescentis*	DSM 20083		no activity (OD) [74]
	*longum*	DSM 20088		no activity (OD) [74]
*Enterococcus*	*faecalis*	ATCC 29212	16.0–17.0 mm [73], 7.9–8.4 mm [76], 5.0–7.0 mm [70], no activity [13]	MIC_50_ = 1/64 (aerial parts), MIC_50_= 1/128 (leaves) [76]
		ATCC 51229		no activity 0.5% solution [56]
		JCM 1513		stimulation of growth (OD) [74]
		clinically isolated strain		no activity [59]
	*faecium*	not specified	14.5–16.0 mm [73], 5.0–7.0 mm [70]	
	*hirae*	ATCC 9790	no activity [13]	
*Lactobacillus*	*rhamnosus*	B-445, ŁOCK 0900		no activity 0.5% solution [56], no activity (OD) [74]
	*plantarum*	299v		no activity 0.5% solution [56]
*Listeria*	*innocua*	ATCC 33090		0.25% solution [56]
	*monocytogenes*	ATCC 19111		0.25% solution [56], no activity [59]
		ATCC 13932	8.2–8.8 mm [76]	MIC_50_ = 1/64 (aerial parts), MIC_50_ = 1/128 (leaves) [76]
		ATCC 15313		no activity 0.5% solution [56]
		ATCC 7644		no activity 0.5% solution [56]
		IFM 1011		no activity 0.5% solution [56]
*Micrococcus*	*luteus*	clinically isolated strain		activity similar to vancomycin [63]
*Staphylococcus*	*aureus*	DSM 20231	4.0–10.0 mm [58]	
		ATCC 12600	11.0–17.0 mm [60], 7.0 mm [61]	
		ATCC 6538P	8.8–10.7 mm [76], 13.5–14.0 mm [64]	325 µg/mL [59], 6.1 mg/mL [64]; MIC_50_ = 1/64 (aerial parts), MIC_50_ = 1/128 (leaves) [76]
		ATCC 6538	24.0 mm [72], 17.5–20.0 mm [68], 10.0 mm (MeOH) [75], 14.0 mm (H_2_O) [75]	0.5 mg/mL [72], 125–250 µg/mL [67], 30% solution [68]
		4.4		0.15% solution [56]
		ATCC 6535		0.15% solution [56]
		ATCC 4538		0.15% solution [56]
		MFBF 124 (MRSA)	14.5 mm [64]	7.6 mg/mL [64]
		ATCC 25923	5.0–21.5 mm [66], 13.3–15.0 mm [71], 7.0 mm [69], activity found; no data [13]	3.13–6.25 mg/mL [13], 156 µg/mL [69], 0.15% solution [56]
		clinically isolated strains		325 µg/mL [59], less active than vancomycin [63]
		MRSA clinically isolated strain	21.0 mm [55]	0.625 µg/mL [55]
		not specified	6.0–7.0 mm [73]	
	*epidermidis*	ATCC 12228	18.2–18.4mm [71]	
*Streptococcus*	*epidermidis*	not specified	6.0–8.5 mm [73]	
	*pneumoniae*	ATCC 49619	16.5–18.0 mm [73], 5.0–7.0 mm [70]	
	*pyogenes*	ATCC 12345		325 µg/mL [59]
		ATCC 19615	14.0–16.4 [71]	-
	*sanguis*	CDC SS 910		325 µg/mL [59]
*Acinetobacter*	*baumannii*	clinically isolated MDR 210	10.0 mm [55]	1.25 µg/mL [55]
		clinically isolated MDR 211	14.0 mm [55]	1.25 µg/mL [55]
*Chromobacterium*	*violaceum*	CV12472	no activity (for QS inhibition) [65]	
*Enterobacter*	*cloacae*	ATCC 23355	no activity [71]	
		PCM 533		no activity [74]
*Escherichia*	*coli*	ATCC 8739	4.0–10.0 mm [58]	
		ATCC 15221		no activity [59]
		ATCC 8677	6.0–7.0 mm [60], no activity [60,61]	
		ATCC 10535	13.5–14.5 mm [64]	6.1–7.6 mg/mL [64]
		ATCC 10536	no activity [76]	62.5–125 µg/mL [67], no activity 0.5% solution [56]
		ATCC 11229	(H_2_O) 11.0 mm [75], (MeOH) 13.0 mm [75]	
		ATCC 35218	no activity [13]	
		MFBF P11 (p-fimbriae positive)	14.5 mm [64]	9.1–16.2 mg/mL [64]
		ATCC 25922	21.0 mm [72], 8.0 mm [69], ≤10 mm [68], 11.5–15.0 mm [66], no activity [13,71]	156 µg/mL [69], 1.0 mg/mL [72], no activity for 30 and 50% solution [68]
		ATCC 25923		no activity 0.5% solution [56]
		PeruMycA 2		125–250 µg/mL [67]
		PeruMycA 3		125–250 µg/mL [67]
		PBio 729 (MRGN)	no activity [75]	(MeOH) 50 µg/mL, (H_2_O) 100 µg/mL [75]
		PBio 730 (MRGN)	no activity [75]	(MeOH) 50 µg/mL, (H_2_O) 100 µg/mL [75]
		ATCC 11239	11.0–13.0 mm [75]	
		clinically isolated MDR 55	16.0 mm [55]	0.625 µg/mL [55]
		clinically isolated strain		more active than tetracycline [63]
		not specified	13.0–15.0 mm [73]	64 µg/mL [74]
*Klebsiella*	*pneumoniae*	ATCC 10031		81 µg/mL [59]
		ATCC 13883	no activity [13,71]	
		MDR 104	14.0 mm [55]	0.625 µg/mL [55]
		MMLRD not specified	13.0–23.5 mm [66]	
*Proteus*	*mirabilis*	MFBF 624	no activity [64]	15.1–16.2 mg/mL [64]
	*vulgaris*	RSKK 96029	activity found; no data [13]	3.13 mg/mL [13]
		ATCC 13315	15.2–17.3 mm [71]	
*Pseudomonas*	*aeruginosa*	ATCC 27853	10.5–11.5 mm [64], 0.0–12.0 mm [75], no activity [13,71,76]	162 µg/mL [59], 9.1 mg/mL [64] no activity [71]
		ATCC 9721	7.0 mm [60], no activity [60,61]	
		PeruMycA 5		62.5–125 µg/mL [67]
		ATCC 2753	4.0–6.0 mm [70]	
		clinically isolated MDR 40	9.0 mm [55]	1.25 µg/mL [55]
		clinically isolated MDR 215	11.0 mm [55]	1.25 µg/mL [55]
		MMLRD not specified	4.0–25.5 mm [66]	
		clinically isolated strain		more active than tetracycline [63]
	*fluorescens*	not specified	6.0 mm [70], 7.0–8.5 mm [73]	
*Salmonella*	*enterica*	ATCC 29631		0.15% solution [56]
	*enteritidis*	IAL 1132		no activity [59]
		ATCC 13076	no activity [76]	
	Hofit	IFM 2318		no activity 0.5% solution [56]
	Typhimurium	ATCC 14028	6.0 mm [69], no activity [13,71]	312 µg/mL [69]
	Typhi	PeruMycA 7		125–250 µg/mL [67]
*Sarcina*	*lutea*	ATCC 9341	15.0–16.5 mm [73]	
*Serratia*	*lutea*	ATCC 9341	8.0–16.0 mm [70]	
	*marcescens*	ATCC 8100	no activity [71]	
		not specified	7.0–15.0 mm [70], 9.0–10.5 mm [73]	
*Shigella*	*flexneri*	CDC 9767		no activity [59]
		IAL 1517		no activity [59]

Abbreviations: ATCC—American Type Culture Collection (USA); NRRL—Agricultural Research Service Culture Collection (USA); NCM—National Centre for Mycology in Edmonton (Canada); DSM: Deutsche SammLung von Microorganismen (Germany); FOMK—Division of Pharmacognosy, University of Helsinki (Finland); YMBL—Division of General Microbiology, University of Helsinki (Finland); OMH—Ontario Ministry of Health, Mycology Laboratory, Toronto, (Canada); OGH—Ottawa General Hospital, Microbiology Laboratory, (Canada); MFBF—collection of the Department of Microbiology, Faculty of Pharmacy and Biochemistry, University of Zagreb (Croatia); CMBC—China General Microbiological Culture Collection Center, Beijing (China); MMLRD—Medical Microbiology Laboratory, Research Hospital of Dicle University in Diyarbakir (Turkey); PeruMycA—culture collection of the Department of Chemistry, Biology and Biotechnology, University of Perugia (Italy); CDC—Collecione de Coltura, IAL- Istituto Alberto Luz (Italy); NCTC—The National Collection of Type Cultures, UK Health Security Agency, (UK). MIC—minimum inhibitory concentration; MBC—minimum bactericidal concentration; MDR—multi-drug resistance, MRSA—methicillin-resistant *S. aureus*; MRGN—Multi-resistant Gram-negative bacilli.

**Table 3:** Antifungal activity of *Epilobium angustifolium* extracts.

Genus	Species	Strains	Inhibition Zone	MIC
*Arthroderma*	*crocatum*	CCF 5300		15.62–31.25 µg/mL [67]
	*currey*	CCF 5207		31.25–62.5 µg/mL [67]
	*gypseum*	CCF 6261		125–250 µg/mL [67]
	*insingulare*	CCF 5417		31.25–62.5 µg/mL [67]
	*quadrifidum*	CCF 5792		62.5–125 µg/mL [67]
*Epidermophyton*	*floccosum*	NCM 335	-	3 mg/mL [62]
		clinically isolated strain		3 mg/mL [62]
*Microsporum*	*canis*	NCM 336		1 mg/mL [62]
		clinically isolated strains		10 µg/mL [59], no growth [62]
	*gypseum*	OMH FR323	18.9 mm [57]	
		clinically isolated strains		650 µg/mL [59], no growth [62]
*Trichophyton*	*erinacei*	CCF 5930		125–250 µg/mL [67]
	*rubrum*	CCF 4879		62.5–125 µg/mL [67]
		CCF 4933		15.62–31.25 µg/mL [67]
		clinically isolated strains		650 µg/mL [59], no growth, 3000 mg/mL [62]
	*mentagrophytes*	OMH T2379	16.1 mm [57]	
		CCF 4823		62.25–125 µg/mL [67]
		clinically isolated strains		162–650 µg/mL [59], 3–6 mg/mL [62]
	*tonsurans*	NCM 334		3 mg/mL [62]
		CCF 4834		7.81–15.62 µg/mL [67]
		clinically isolated strain		no growth [62]
*Aspergillus*	*flavus*	clinically isolated strains		no activity [62]
	*fumigatus*	OMH FR2837	no activity [57]	
		NCM 338		no activity [62]
		clinically isolated strains		no activity [62]
	*niger*	ATCC 16404	no activity [58]	
*Candida*	*albicans*	ATCC 10231	15.0–20.0 mm [60], 16.0 mm [72], 10.0–11.7 mm [76], 1.0–3.0 mm [58], no activity [13,61,64]	6.1–12.1 mg/mL [64], 4 mg/mL [72] MIC_50_ = 1/64 (leaves), MIC_50_ = 1/32 [76]
		ATCC 24433		0.8 mg/mL [62]
		ATCC 90028		0.4 mg/mL [62]
*Candida*	*albicans*	ATCC 885–653	10.0–15.0 mm [68]	
		OGH 308–1329	13.5 mm [57]	
		DBVPG 6379		125–250 µg/mL [67]
		DBVPG 6183		125–250 µg/mL [67]
		clinically isolated strains		0.2–1.6 mg/mL [62], 325 µg/mL [59]
	*dubliniensis*	MFBF 501	no activity [64]	12.1 mg/mL [64]
	*glabrata*	clinically isolated strains		25–50 mg/mL [62], no activity [59]
	*krusei*	ATCC 6258	no activity [13]	0.2 mg/mL [62]
		clinically isolated strains		0.2 mg/mL [62], 325 µg/mL [59]
	*maltosa*	SBUG 700	18.0 mm (H_2_O), 25 mm (MeOH), no activity (DCM) [75]	
	*lusitaniae*	clinically isolated strains		0.05–0.2 mg/mL [62]
	*parapsilosis*	ATCC 90018		0.8 mg/mL [62]
		ATCC 22019		0.1 mg/mL [62]
		DBVPG 6551		≥250 µg/mL [67]
		clinically isolated strains		0.1–0.4 mg/mL [62]
	*tropicalis*	ATCC 750	no activity [64]	1.6 mg/mL [62], 15.2 mg/mL [64]
		Y-12968	no activity [13]	
		DBVPG 6184		31.25–62.5 µg/mL [67]
		clinically isolated strain		1.6 mg/mL [62]
	spp.	ATCC 885–653	≤10.0–15.0 mm [68]	30% solution [68]
*Saccharomyces*	*cerevisiae*	ATCC 48252	15.5 mm [57]	
		NCYC 87	no activity [64]	18.2 mg/mL [64]
		clinically isolated strains		0.05 mg/mL [62]
*Cryptococcus*	*neoformans*	OMH FR2704	11.5 mm [57]	
		clinically isolated strains		0.4 mg/mL [62]
*Fusarium*	*solani*	Pig pasture soil		no activity [62]
*Rhizopus*	sp.	NCM 337		25 mg/mL [62]
		clinically isolated strain		0.2 mg/mL [62]

Abbreviations: ATCC—American Type Culture Collection, Rockville (USA); NRRL—Agricultural Research Service Culture Collection (USA); NCM—National Centre for Mycology in Edmonton (Canada); CCF—Culture Collection of Fungi, Department of Botany, Charles University, Prague (Czech Republic); DSM—Deutsche Sammlung von Microorganismen (Germany); OMH—Ontario Ministry of Health, Mycology Laboratory, Toronto (Canada); OGH—Ottawa General Hospital, Microbiology Laboratory (Canada); MFBF—collection of the Department of Microbiology, Faculty of Pharmacy and Biochemistry, University of Zagreb (Croatia); SBUG—Department of Biology, University of Greifswald (Germany); NCYC—National Collection of Yeast Cultures (UK); H_2_O—aqueous extract; MeOH—methanol extract; DCM—dichloromethane extract.

## Data Availability

Not applicable.
